# Outcomes of a PrEP Demonstration Project with LGBTQ Youth in a Community-Based Clinic Setting with Integrated Gender-Affirming Care

**DOI:** 10.1089/trgh.2019.0069

**Published:** 2020-06-08

**Authors:** Maureen D. Connolly, Doreen N. Dankerlui, Tony Eljallad, Isadore Dodard-Friedman, Amy Tang, Christine L.M. Joseph

**Affiliations:** ^1^ Department of Pediatrics, Henry Ford Health System, Detroit, Michigan, USA.; ^2^ Global Health Initiative, Henry Ford Health System, Detroit, Michigan, USA.; ^3^ Department of Internal Medicine, University of Utah School of Medicine, Salt Lake City, Utah, USA.; ^4^ Department of Public Health Sciences, Henry Ford Health System, Detroit, Michigan, USA.

**Keywords:** pre-exposure prophylaxis, LGBT, transgender, youth, community-based

## Abstract

Lesbian, gay, bisexual, transgender, and queer/questioning (LGBTQ) youth are disproportionately affected by HIV, and young transgender women (YTW) are especially impacted. The purpose of this small demonstration project was to measure pre-exposure prophylaxis (PrEP) adherence in a community-based clinic for LGBTQ youth in which PrEP services are integrated with gender-affirming care. Of the 50 enrolled participants, 38 had a serum drug assay performed after three or more months and 26% of those had laboratory evidence of highly protective levels of medication. Low adherence highlights the need for culturally tailored follow-up efforts and assistance with the structural barriers to health experienced by LGBTQ youth, especially YTW.

## Introduction

Lesbian, gay, bisexual, transgender, and queer/questioning (LGBTQ) youth are disproportionately affected by HIV^1^ and young transgender women (YTW) are especially impacted.^2^ In Metropolitan Detroit, which accounts for nearly two-thirds of Michigan's HIV prevalence, 51% of new diagnoses occurred in people ages 13–30 years in 2018, the majority of which were in young cisgender men who have sex with men (YCMSM) and YTW.^3^ Tenofovir-emtricitabine (TDF-FTC)-based pre-exposure prophylaxis (PrEP) is highly effective in preventing HIV^4^ and is safe for use in adolescents and young adults,^5,6^ but there is still limited knowledge about effective real-world implementation of PrEP for LGBTQ youth. Barriers to accessing PrEP include inadequate health insurance, a limited number of LGBTQ-affirming providers, residential instability, and economic marginalization.^7–9^ In addition, there is a growing understanding of the need to include gender-affirming care in any effort to prevent or treat HIV within the LGBTQ community.^10,11^

To better serve this population, a collaboration developed in 2016 between Henry Ford Health System (HFHS), a major health system in Southeastern Michigan, and the Ruth Ellis Center, a community-based organization that supports Detroit-area runaway, homeless, and at-risk LGBTQ youth. This partnership led to the development of an LGBTQ health and wellness program that provides community-based medical care, behavioral health, and case management services to young people ages 10–30 years. Medical services include gender-affirming hormones, PrEP, HIV treatment, and general primary care. There is no out-of-pocket cost for visits, tests, and medications, including hormones, which are delivered on-site and picked up by patients during their visit. The clinic is colocated with a drop-in center where youth can access hot meals, food boxes, clothing, showers, laundry, computers, a dance studio, and space for socialization. This collaboration has enabled providers to engage some of the most marginalized members of the Detroit LGBTQ community in discussions of PrEP, especially transgender women.^12^ YTW represent roughly 50% of the PrEP-eligible patients seen at the clinic, whereas the remainder are either YCMSM or young transgender men who have sex with men (YTMSM). The purpose of this demonstration project was to describe PrEP use among LGBTQ youth in a community-based setting with integrated no-cost gender-affirming care.

## Methods

Data were collected between November 2017 and December 2018. Individuals were eligible to participate if they were between 18 and 30 years; identified as YTW, YTMSM, or YCMSM; were currently taking or wanted to start PrEP; had a nonreactive HIV test; no laboratory evidence of hepatitis B or C infection, and normal creatine clearance (>60 mL/min). This study was approved by the HFHS Institutional Review Board and informed consent was obtained from all participants.

Individuals were enrolled upon presentation for clinical care (index visit) and followed monthly for three additional visits. After routine clinical practice, providers collected medical and social history at each visit, using a standardized electronic medical record (EMR) template developed for use at Ruth Ellis that allowed for documentation of patient response by selecting from predetermined options. Laboratories done at the initial and final follow-up visits were completed through normal clinic protocol and included rapid and laboratory-based fourth-generation HIV, rapid plasma regain (RPR), creatinine, hepatitis B and C serologies, and gonorrhea and chlamydia RNA testing from urine, rectum, and pharynx. In addition, a TDF assay using dried blood spots (DBS) was collected once at the participant's last follow-up visit and sent to an external laboratory for processing. There were no costs for services or medication (standard clinic practice) and there were no additional incentives offered for participation.

### Measures

The primary outcome of PrEP adherence was measured through TDF DBS once at the final follow-up visit (defined as the last visit between 3 and 6 months postenrollment). “Highly protective” was defined as a TDF DBS value ≥700 fmol/punch, consistent with ≥4 doses of TDF-FTC per week and “somewhat protective” was defined as a TDF DBS value ≥350 fmol/punch, consistent with ≥2 doses of TDF-FTC per week.^13,14^ Participant-reported adherence was also collected using a three-item validated measure administered verbally by the provider during each visit.^15^

### Analysis

Patient demographic and laboratory data were retrieved from the EMR. Natural Language Programming (NLP) was used to extract social and medical history data from the study templates completed by providers during each visit. Patient characteristics were examined overall and by PrEP adherence status. Associations were evaluated using relative risks (RRs) and corresponding 95% confidence intervals (CIs) and *p*-values, with statistical significance defined as *p*<0.05. All analyses were performed using SAS version 9.4 (SAS Institute, Cary, NC).

## Results

Of the 80 PrEP-eligible patients seen during the study period, 50 (62.5%) chose to start or continue PrEP and participate in the study. Of the 50 initially enrolled (Table 1), 78% identified as black/African American, 14% as white, 4% as Hispanic/Latinx, and 6% as Arab American. Half (50%) of the participants identified as transgender women, 38% as cisgender men, and 12% as transgender men. Participant median age was 26 years (range 18–29).

**Table 1.** Demographics of Participants at Baseline (*n*=50)

Age, years	
Median (range)	26 (18–29)
Race/ethnicity, *n* (%)	
Black/African American	39 (78)
White	7 (14)
Hispanic/Latinx	2 (4)
Arab American	3 (6)
Education, *n* (%)	
High school/GED—no	13 (26)
High school/GED—yes	33 (66)
College degree	4 (8)
Housing—number of places slept, *n* (%)	
1–2 places	40 (80)
≥3 places	10 (20)
Gender identity, *n* (%)	
Cisgender man	19 (38)
Transgender woman	25 (50)
Transgender man	6 (12)
Gender affirming hormones use, past or present, *n* (%)	
Yes	31 (62)
No	19 (38)
Self-reported history of STI, *n* (%)	
Yes	23 (46)
No	27 (54)
Positive STI laboratory result, *n* (%)	
Yes	12 (24)
No	38 (76)
Condom use, *n* (%)	
Always	14 (28)
Sometimes	32 (64)
Never	4 (8)
Commercial sex work, *n* (%)	
Yes	14 (28)
No	36 (72)
Smokes—cigarettes, *n* (%)	
Everyday	16 (32)
Sometimes	11 (22)
No	23 (46)
Smokes—marijuana, *n* (%)	
Everyday	23 (46)
Sometimes	9 (18)
No	18 (36)
Uses alcohol—five or more drinks, *n* (%)	
Everyday	1 (2)
Sometimes	20 (40)
No	29 (58)
Uses other drugs, *n* (%)	
Yes	5 (10)
No	45 (90)

GED, General Educational Development/Diploma; STI, sexually transmitted infection.

At the initial visit, 23 patients (46%) reported a history of sexually transmitted infection (STI) and 12 participants (24%) had one or more positive STI results, including gonorrhea (rectal *n*=2, pharyngeal *n*=1, urine *n*=1), rectal chlamydia (*n*=5), and RPR (*n*=7). Although there were no HIV seroconversions during the study, one enrolled individual was subsequently withdrawn from the sample after a negative rapid HIV test at enrollment was followed by a positive laboratory result 5 days later. Of the remaining 49 participants, 78% (*n*=43) completed at least one follow-up visit and 37% (*n*=18) completed three monthly follow-up visits.

### Adherence

Of the 43 participants completing ≥1 follow-up visit, TDF assays were obtained for 38 at a final visit (3–6 months postenrollment). According to assay results, 10 (26%) participants achieved protective status, and an additional 5 (13%) somewhat protective status. Twenty-seven participants (71%) reported having taken ≥4 doses a week (Table 2). Table 3 presents RR and 95% CIs describing the relationship between patient-level factors and TDF assay-determined PrEP adherence. Smoking cigarettes, RR=0.12 (0.12–0.88), *p*=0.009 and smoking marijuana, RR=0.29 (0.11–0.76), *p*=0.008, were significantly inversely associated with adherence. Stable housing was positively associated with adherence and had a notable RR=4.1 (0.59–33.3), although the *p*-value was >0.05.

**Table 2.** Measured and Self-Reported Adherence

Measured adherence		
≥700	≥350–699	<350
10 (26%)	5 (13%)	23 (61%)
Self-reported adherence		
>16 days of dose taken	≥8–12 days of dose taken	<8 days of dose taken
27 (71%)	2 (5%)	9 (24%)

Total *N*=38.

**Table 3.** Associations Between Sociodemographic Variables and Measured Adherence at Visit 4

	Tenofovir level ≥700 (n=10)	Tenofovir level <700 (n=28)	Total (N=38)	RR	95% CI	p^a^
Sociodemographics						
Age, *n* (%)						
27–30	5 (41.6)	7 (58.3)	12	Reference		
23–26	1 (7.1)	13 (92.9)	14	0.17	0.02–1.27	0.065
18–22	4 (33.3)	8 (66.7)	12	0.80	0.28–2.27	0.99
Race/ethnicity, *n* (%)						
White	2 (33.3)	4 (66.7)	6	Reference		
Black or African American	6 (21.4)	22 (78.6)	28	0.64	0.17–2.44	0.61
Other^b^	2 (50.0)	2 (50.0)	4	1.50	0.34–6.70	0.99
Education, *n* (%)						
High school/GED—yes	8 (29.6)	19 (70.4)	27	1.64	0.41–6.67	0.69
High school/GED—no	2 (18.2)	9 (81.8)	11			
Housing (no. of places slept), *n* (%)						
1–2 places	9 (34.6)	17 (65.4)	26	4.17	0.59–33.3	0.12
≥3 places	1 (8.3)	11 (91.7)	12			
Gender related						
Gender identity, *n* (%)						
Cisgender man	5 (33.3)	10 (66.6)	15	Reference		
Transgender woman	4 (21.1)	15 (78.9)	19	0.63	0.20–1.95	0.46
Transgender man	1 (25.0)	3 (75.0)	4	0.75	0.12–4.73	0.99
Gender affirming hormones use, past or present, *n* (%)						
Yes	5 (21.7)	18 (78.3)	23	0.65	1.35–11.0	0.47
No	5 (33.3)	10 (66.7)	15			
Sexual health						
Condom use, *n* (%)						
Always/sometimes	8 (24.2)	25 (75.8)	33	0.60	0.18–1.2	0.59
Never	2 (40.0)	3 (60.0)	5			
Commercial sex work, *n* (%)						
Yes	3 (30.0)	7 (70.0)	10	1.2	0.38–3.70	0.99
No	7 (25.0)	21 (75.0)	28			
Substance use						
Smokes—cigarettes						
Everyday/sometimes	1 (5.6)	17 (94.4)	18	0.12	0.02–0.88	0.009
No	9 (45.0)	11 (55.0)	20			
Smokes—marijuana						
Everyday/sometimes	4 (18.2	18 (81.8)	22	0.29	0.11–0.76	0.008
No	10 (62.5)	6 (37.5)	16			
Uses alcohol—five or more drinks						
Everyday/sometimes	3 (25.0)	9 (75.0)	9	0.93	0.29–2.94	0.99
No	7 (26.9)	19 (73.1)	26			
Uses other drugs						
Yes	1 (14.3)	6 (85.7)	7	0.49	0.07–3.2	0.65
No	9 (29.0)	22 (71.0)	31			

^a^ Fisher's exact test.

^b^ Includes *n*=1 Hispanic/Latinx patient and *n*=3 Arab-American patients.

CI, confidence interval; RR, relative risk.

## Discussion

Through a community-based approach that includes accessible gender-affirming care, we engaged LGBTQ youth, specifically YTW, in PrEP services. This setting allowed providers to address barriers to PrEP access that have been previously described, such as high cost and provider hesitation to discuss PrEP and/or gender identity.^16–18^ Despite providing services in a setting specifically designed for LGBTQ youth, both retention and adherence were suboptimal. This is consistent with other studies of PrEP adherence among transgender women^19^ and young men who have sex with men^5,6^ in which a majority of participants did not have evidence of highly protective drug levels over time.

The discrepancy between measured adherence (26%) and self-reported adherence (71%) may be due to recall bias and to social desirability bias since the adherence tool was administered by the providers.^20^ These biases may be especially pronounced in young adults and laboratory-based measures of drug levels will be vital to gaining an accurate understanding of medication adherence in this population.

Both smoking cigarettes and marijuana were significantly inversely associated with measured adherence. With marijuana use on the rise,^21^ it will be important to examine how this impacts PrEP use. Young people may use it to self-medicate for underlying anxiety, depression, or post-traumatic stress disorder.^22–24^ Increasing access to effective mental health treatment modalities to supplant or supplement marijuana may be important to supporting medication adherence among LGBTQ youth in the future.

### Limitations

This was a small study in which participants were recruited while seeking clinical care within an organization that specifically serves LGBTQ youth, and findings may not be representative of young people seeking care in more traditional health care settings. Owing to the limited number of participants with a TDF assay (*n*=38), we were only able to examine PrEP adherence in a small sample. Real-world adherence studies with larger samples of LGBTQ youth, especially YTW, are necessary to further understand barriers and facilitators to PrEP utilization.

## Conclusion

Despite these limitations, we believe our results contribute to emerging research about PrEP utilization among LGBTQ youth. A community-based setting that provides wraparound services, including no-cost gender-affirming care, was used to engage young people previously under-represented in PrEP research. To our knowledge, this is the first study of real-world PrEP adherence in which the largest subgroup of participants were young transgender people of color.

Low measured levels of TDF demonstrate the need for culturally tailored follow-up efforts, such as peer navigator services or support groups led by members of the transgender community. In addition, adherence support should include assistance with the structural barriers to health that LGBTQ youth, especially transgender youth, experience outside of medical settings, including assistance with identification documents, transportation, and navigation of housing/shelter services that are affirming to transgender residents.^25^ Future research on the role these factors play in PrEP adherence is vital to supporting a group of young people who are disproportionately impacted by HIV.

## Abbreviations Used

CI: confidence interval
DBS: dried blood spots
EMR: electronic medical record
GED: General Educational Development/Diploma
HFHS: Henry Ford Health System
NLP: Natural Language Programming
PrEP: pre-exposure prophylaxis
RPR: rapid plasma regain
RR: relative risk
STI: sexually transmitted infection
TDF-FTC: tenofovir-emtricitabine
YCMSM: young cisgender men who have sex with men
YTMSM: young transgender men who have sex with men
YTW: young transgender women
